# Dairy Cows Experimentally Infected With Bovine Leukemia Virus Showed an Increased Milk Production in Lactation Numbers 3–4: A 4-Year Longitudinal Study

**DOI:** 10.3389/fmicb.2022.946463

**Published:** 2022-07-11

**Authors:** Yi Yang, Zaicheng Gong, Yi Lu, Xubin Lu, Jilei Zhang, Ye Meng, Yalan Peng, Shuangfeng Chu, Wenqiang Cao, Xiaoli Hao, Jie Sun, Heng Wang, Aijian Qin, Chengming Wang, Shaobin Shang, Zhangping Yang

**Affiliations:** ^1^Jiangsu Co-innovation Center for Prevention and Control of Important Animal Infectious Diseases and Zoonoses, College of Veterinary Medicine, Yangzhou University, Yangzhou, China; ^2^International Corporation Laboratory of Agriculture and Agricultural Products Safety, Yangzhou University, Yangzhou, China; ^3^College of Animal Science and Technology, Yangzhou University, Yangzhou, China; ^4^Division of Gastroenterology and Hepatology, Department of Medicine, University of Illinois at Chicago, Chicago, IL, United States; ^5^Shenzhen Academy of Inspection and Quarantine Sciences, Shenzhen, China; ^6^Department of Pathobiology, College of Veterinary Medicine, Auburn University, Auburn, AL, United States

**Keywords:** bovine leukemia virus, artificially inoculation, milk yield, somatic cell score, nutrition compositions

## Abstract

Bovine leukemia virus (BLV) is widespread in global cattle populations, but the effects of its infection on milk quantity and quality have not been clearly elucidated in animal models. In this study, 30 healthy first-lactation cows were selected from ≈2,988 cows in a BLV-free farm with the same criteria of parity, age, lactation number, as well as milk yield, SCS, and composition (fat, protein, and lactose). Subsequently, these cows were randomly assigned to the intervention (*n* = 15) or control (*n* = 15) group, and reared in different cowsheds. Cows in the intervention group were inoculated with 1 × phosphate-buffered solution (PBS) resuspended in peripheral blood mononuclear cells (PBMC) from a BLV-positive cow, while the controls were inoculated with the inactivated PBMC from the same individual. From June 2016 to July 2021, milk weight (kg) was automatically recorded by milk sensors, and milk SCS and composition were originated from monthly performed dairy herd improvement (DHI) testing. Fluorescence resonance energy transfer (FRET)–qPCR and ELISA showed that cows in the intervention group were successfully infected with BLV, while cows in the control group were free of BLV for the entire period. At 45 days post-inoculation (DPI), the numbers of whole blood cells (WBCs) (*P* = 0.010), lymphocytes (LYMs) (*P* = 0.002), and monocytes (MNCs) (*P* = 0.001) and the expression levels of IFN-γ (*P* = 0.013), IL-10 (*P* = 0.031), and IL-12p70 (*P* = 0.008) increased significantly in the BLV infected cows compared to the non-infected. In lactation numbers 2–4, the intervention group had significantly higher overall milk yield (*P* < 0.001), fat (*P* = 0.031), and protein (*P* = 0.050) than the control group, while milk SCS (*P* = 0.038) and lactose (*P* = 0.036) decreased significantly. Further analysis indicated that BLV infection was associated with increased milk yield at each lactation stage in lactation numbers 3–4 (*P* = 0.021 or *P* < 0.001), but not with SCS and milk composition. Together, this 4-year longitudinal study revealed that artificial inoculation of BLV increased the milk yield in cows in this BLV challenge model.

## Introduction

Bovine leukemia virus (BLV) is a member of *deltaretroviruses* evolutionarily close to human T-cell leukemia virus type 1 (HTLV-1), causing enzootic bovine leucosis (EBL) (Aida et al., 2013). Although it is a high level of infection, more than 95% of infected cows will not develop B-cell lymphoma, while ≈30% of them will suffer from non-malignant proliferation of untransformed B lymphocytes, termed persistent lymphocytosis, and fewer than 5% develop malignant lymphosarcoma after a long incubation period (Bartlett et al., 2013). These tumors can reduce an animal welfare and lead to premature death. Therefore, the possible effects of BLV infection on dairy cattle, including reduced milk yield, increased somatic cell count (SCC), and early slaughter, have attracted more attention (Erskine et al., 2012; Bartlett et al., 2013) because the positive rates are higher with the extension of feeding time than those in beef cattle. In the absence of an effective treatment and commercial vaccines, quarantine and early slaughter are the only feasible ways to control the transmission of BLV (Kuczewski et al., 2021). BLV was only eradicated from some Western European countries, Australia, and New Zealand and was endemic in other countries, including China, Brazil, and the United States (LaDronka et al., 2018; Ma et al., 2021; Ramalho et al., 2021). In contrast to the situation of Western European countries, within-herd and between-herd prevalences of BLV in Asian, North American, and South American countries were too high to be controlled through eradication programs (Kobayashi et al., 2010; Nekouei et al., 2015a; Ramalho et al., 2021).

BLV naturally infects cattle (*Bos taurus* and *Bos indicus*), buffaloes (*Bubalus bubalis*), and capybaras (*Hydrochoerus hydrochaeris*) and can experimentally challenge and generate an immune response in a wide range of animal species, including sheep, goat, pig, rabbit, rat, and chicken (Mammerickx et al., 1981; Olson et al., 1981; Altanerova et al., 1990; Kucerova et al., 1999; Krasnikova et al., 2019; Porta et al., 2019). Among these animals, sheep was most commonly employed as they develop tumors earlier and more frequently following challenge (Kuczewski et al., 2021). Most of the studies carried out the cytodynamics in BLV-infected ewes as a model; nonetheless, there are significant differences between cattle and ewes in the response to BLV infection (Florins et al., 2007). Therefore, due to the differences in the genetic background, the naturally infected hosts, dairy and beef cattle, are considered to be the best model to study the immune response, viral persistence, and pathogenesis in BLV infection. Although several previous studies have described the dynamics and transmission of BLV after experimental inoculation (Benitez et al., 2019; Hutchinson et al., 2020), to the best of our knowledge, there have been no studies so far to evaluate the effect of BLV on the dairy performance of cows in artificial inoculation models. Hence, we designed and implemented the following experiments to evaluate whether BLV infection has effects on dairy performance of cows.

## Materials and Methods

### Selection of Experimental Cows

This study was performed in a BLV-negative dairy farm in Jiangsu province, China. At the beginning of this study (June 2017), the farm had a total number of 2,988 cows, of which 2,898 were lactating cows, including 2,222 (76.67%) of first-lactation cows, 337 (11.63%) of second-lactation cows, and 339 (11.70%) of ≥third-lactation cows. The overall average daily milk yield of the farm was 26.6 kg/d, while the first-, second-, and ≥ third-lactation cows had average daily milk yields of 24.5, 32.6, and 33.8 kg/d, respectively. The average days in milk (DIM), voluntary waiting period, and calving interval were 247, ≈50, and 368 d, respectively. The cows in this farm had an average age of 22.83 months at first calving. The value of a 21-day pregnancy rate is 21.67%, while 25.8% of cows were not pregnant at 150 DIM. The cows were provided with standard diet formulation, comfortable housing, and adequate water (Erickson and Kalscheur, 2020).

It is well known that the performance of individual dairy cattle varies with the genetic background of cows, parity, lactation stages, seasons, and feed ingredients. To minimize the impact of factors, other than BLV infection, on dairy performance, we analyzed the pedigree, milk yield, and dairy herd improvement (DHI) records and comprehensively selected 30 cows from 2,988 candidates in the dairy farm in Jiangsu province, China. These enrolled Holstein dairy cattle were all in the same lactation number with similar weight (6-month-old weight), individual performance, and predicted calving date and randomly divided into intervention (*n* = 15) and control (*n* = 15) groups, respectively, using a random number generator function (Table 1). The sample size of 15 cows in each group can effectively reduce the error within the group without violating the requirements of animal ethics guidelines due to the abuse and waste of the number of animals and can meet the requirement of the subsequent statistical analysis (White et al., 2010). There were no significant differences in milk yield, SCS, and the contents of fat, protein, and lactose between the two groups of cows before inoculation (Table 1). All the experimental cows were determined to be free of BLV and some other pathogens, including bovine herpesvirus 1, bovine viral diarrhea virus, *Mycobacterium bovis, Brucella abortus, Anaplasma* spp., *Babesia* spp., and *Theileria* spp. by in-house fluorescence resonance energy transfer (FRET)–qPCRs or commercial ELISAs (Yang et al., 2014, 2018; Li et al., 2015; Johnson et al., 2020).

**Table 1:** Comparison of age, average daily milk yield, SCS, milk composition, predicted calving dates, and culling events of cows in the intervention and control groups before BLV inoculation.

**Cow #**	**Age,** **month^a^**	**Milk yield, kg/d**	**SCS**	**Fat, %**	**Protein, %**	**Lactose, %**	**Predicted calving date^b^**	**Culling**		
								**Date**	**Lactation number**	**reason**
**Intervention group**										
649	45	18.20 ± 5.54^c^	2.00 ± 2.31	4.72 ± 0.68	3.68 ± 0.35	4.98 ± 0.26	06/30	Alive		
2723	45	21.64 ± 5.90	2.00 ± 1.77	4.29 ± 0.91	3.63 ± 0.38	5.14 ± 0.16	07/14	Alive		
5525	44	18.10 ± 4.68	1.67 ± 1.63	4.39 ± 0.30	3.63 ± 0.38	5.14 ± 0.16	07/17	07/31/2017	2	Heat stress
5935	44	22.61 ± 4.92	0.50 ± 0.58	3.36 ± 0.25	3.31 ± 0.02	5.32 ± 0.07	06/26	Alive		
6094	44	18.53 ± 5.86	1.20 ± 1.64	4.03 ± 0.84	3.36 ± 0.12	5.17 ± 0.08	07/18	08/10/2017	1–2	Reproductive syndrome
6771	44	18.62 ± 4.68	1.75 ± 1.58	3.45 ± 0.27	3.19 ± 0.13	5.03 ± 0.21	07/28	01/28/2018	2–3	Pneumonia
16695	43	20.98 ± 6.26	3.60 ± 2.88	4.86 ± 0.56	3.64 ± 0.29	4.88 ± 0.10	07/19	07/18/2017	1–2	Heat stress
17732	42	19.79 ± 5.62	0.83 ± 0.75	3.67 ± 0.76	3.29 ± 0.43	5.10 ± 0.17	06/22	08/15/2019	3–4	Diarrhea
17741	42	21.37 ± 7.13	1.00 ± 1.00	4.29 ± 1.91	3.80 ± 0.62	5.02 ± 0.06	07/16	Alive		
17985	42	18.97 ± 5.10	0.83 ± 0.98	4.29 ± 0.70	3.67 ± 0.52	5.13 ± 0.19	07/21	Alive		
19102	42	17.43 ± 6.23	4.00 ± 2.24	3.70 ± 0.88	3.30 ± 0.64	5.03 ± 0.16	07/17	08/06/2018	3	Diarrhea
19197	42	23.30 ± 5.15	2.60 ± 2.07	3.76 ± 0.70	3.59 ± 0.61	4.89 ± 0.15	07/28	08/15/2017	1–2	Ketosis
19357	42	17.08 ± 5.28	3.00 ± 1.41	3.97 ± 0.13	3.51 ± 0.47	4.84 ± 0.16	07/25	Alive		
19770	42	20.64 ± 7.34	0.00 ± 0.00	NA	NA	NA	07/21	01/28/2018	2–3	Dermatitis verrucosa
20202	42	18.05 ± 4.97	1.60 ± 1.52	4.07 ± 1.14	3.61 ± 0.79	5.05 ± 0.11	07/11	07/15/2017	1–2	Heat stress
Total	43.00 ± 1.20	19.74 ± 5.98	1.75 ± 1.85	4.09 ± 0.84	3.53 ± 0.46	5.04 ± 0.18				
**Control group**										
827	45	19.09 ± 6.00	1.83 ± 1.47	4.24 ± 0.66	3.83 ± 0.41	4.92 ± 0.09	07/28	10/04/2017	2	Ketosis
991	45	20.64 ± 4.89	3.00 ± 2.52	4.76 ± 0.68	3.47 ± 0.29	5.02 ± 0.10	06/23	Alive		
1852	45	19.93 ± 6.31	1.13 ± 1.55	4.20 ± 2.67	3.06 ± 1.80	4.93 ± 0.15	07/16	11/28/2020	4–5	Reproductive syndrome
2520	45	19.49 ± 4.86	3.22 ± 2.17	3.78 ± 0.54	3.43 ± 0.64	4.95 ± 0.10	07/25	06/05/2018	2–3	Pneumonia
3236	44	17.81 ± 6.10	1.20 ± 1.30	3.82 ± 1.00	3.67 ± 0.40	4.80 ± 0.13	07/25	Alive		
3395	44	19.46 ± 4.37	2.00 ± 1.41	4.35 ± 0.61	3.36 ± 0.34	5.10 ± 0.11	07/04	01/27/2018	2	Reproductive syndrome
3593	44	18.04 ± 6.57	2.56 ± 2.13	3.75 ± 0.73	3.54 ± 0.38	5.09 ± 0.35	07/28	08/27/2017	1-2	Heat stress
7533	43	16.92 ± 7.59	1.67 ± 1.37	3.71 ± 0.29	3.22 ± 0.32	4.96 ± 0.10	07/23	03/08/2019	2-3	Dermatitis verrucosa
7965	43	20.16 ± 6.22	2.67 ± 0.58	3.52 ± 0.18	3.58 ± 0.15	5.00 ± 0.12	07/08	09/22/2017	2	Mastitis
8351	43	18.72 ± 4.99	2.20 ± 2.77	3.88 ± 0.33	3.42 ± 0.43	5.09 ± 0.18	07/24	Alive		
8731	43	18.08 ± 6.51	1.33 ± 1.21	3.38 ± 0.30	3.42 ± 0.23	5.22 ± 0.12	07/12	07/23/2020	3	Ketosis
8906	43	19.92 ± 4.98	2.67 ± 1.58	4.17 ± 0.52	3.44 ± 0.45	5.13 ± 0.18	07/22	Alive		
17052	42	22.21 ± 5.24	2.25 ± 1.39	4.58 ± 0.51	3.85 ± 0.40	5.00 ± 0.30	07/26	01/03/2020	4	Dermatitis verrucosa
18272	42	21.75 ± 5.60	1.75 ± 2.43	4.18 ± 0.71	3.17 ± 0.39	5.47 ± 0.12	07/21	Alive		
18452	42	21.21 ± 4.19	1.71 ± 0.95	4.08 ± 0.95	3.65 ± 0.47	5.04 ± 0.18	07/14	08/25/2018	3	Diarrhea
Total	43.53 ± 1.13	19.60 ± 5.86	2.12 ± 1.79	4.08 ± 0.89	3.47 ± 0.58	5.05 ± 0.21				
**Statistical analysis between the intervention and control groups**										
*P*-value	0.219	0.254	0.180	0.940	0.506	0.760				

a *Age at which these cows were experimentally infected*.

b *Cows enrolled in this study were all in their first lactation, and the predicted calving dates of all these cows were in 2016*.

c *Data are shown as mean ± SD*.

After inoculation, the two groups of cows were reared separately in two cowsheds, together with ≈100 cows in each cowshed. These two cowsheds had similar context and were located in the same farm separated by ≈200 m distance. The cows in the two sheds were fed exactly the same diet by the same caregivers, who were not aware of the infection status of these cows, but they did not share the milking machines. Then the raw data of milk yield, SCS, and composition (primary objectives), as well as immune cells and immune-related cytokines in peripheral blood (secondary objectives), were collected and managed by an administrator who was unaware of the infection status of these cows, either.

### Artificially Inoculating Cows With BLV

Sterile whole blood was collected from a BLV-positive (B2145) cow with the combination of dipotassium ethylenediaminetetraacetic acid (EDTA), and proviral load (PVL) was determined by BLV FRET-qPCR, as described previously (Yang et al., 2016b). Subsequently, peripheral blood mononuclear cells (PBMC) were purified and resuspended in sterile 1 × phosphate-buffered solution (PBS) buffer at a concentration of 3.0 × 10^5^ cells/ml. Subsequently, half aliquot of the PBMC was inactivated by incubating at 60°C for 30 min. At the time of inoculation, each cow received a dose of 3.0 × 10^6^ activated (containing 2.7 × 10^6^ BLV proviral copies) or inactivated PBMC *via* intravenous inoculation through the jugular vein. The inoculations were performed by two veterinarians who were not aware of the serial numbers (ear tags were covered) of the cows and the composition of the injection (labeled A or B, respectively).

### Collection of Blood Samples

Whole blood samples were collected from the employed cows before and after inoculation. In detail, two specifications (2 and 10 ml) of evacuated tubes containing EDTA (BD, Franklin Lakes, USA) were used to hold the whole blood samples collected from all the 30 cows at 15 days before inoculation and 0 (2 h), 5, 15, 45, 75, 105, 135, and 165 days post-inoculation (DPI). The samples in the 2-ml tubes were applied for routine complete blood counts and DNA extraction, while the blood in the 10-ml tubes were used for plasma separation. In addition, the control cows were tested every 6 months to ensure that they were free of BLV. Within 2 h, these samples were smoothly transported on ice to the laboratory in Yangzhou University.

### BLV FRET-QPCR and ELISA

DNA was extracted from 200 μl of whole-blood samples using a Roche High Pure PCR Template Preparation Kit (Roche, Mannheim, Germany) in accordance with the manufacturer's instructions. The DNA samples were then stored at −20°C or directly used for analyzes. The FRET-qPCR targeting BLV *pol* gene was applied to screen BLV provirus, as described previously (sensibility: 1 copy/reaction) (Yang et al., 2016a).

Plasma was separated from whole blood samples by centrifugation at 1,500 × g for 20 min. The plasma samples were then stored at −20°C or directly used for analysis. The commercial INgezim BLV Compac 2.0 blocking ELISA kit (Ingenasa, Madrid, Spain) was also used to detect the specific antibodies to BLV gp51 protein following the manufacturer's instructions.

### Routine Complete Blood Counts (CBCs)

Routine CBC was performed on a BC-2800 Vet (Mindray, Shenzhen, China) to quantify 18 parameters, including the number of white blood cells (WBCs) (sensibility: 1 × 10^8^ cells/L), lymphocytes (LYMs) (sensibility: 1 × 10^8^ cells/L), monocytes (MNCs) (sensibility: 1 × 10^8^ cells/L), granulocytes (GRANs) (sensibility: 1 × 10^8^ cells/L), red blood cells (RBC) (sensibility: 1 × 10^10^ cells/L), and platelets (PLT) (sensibility: 1 × 10^9^ cells/L); the percentages of lymphocytes (LYM%) (sensibility: 0.1%), monocytes (MNC%) (sensibility: 0.1%), granulocytes (GRAN%) (sensibility: 0.1%), hematocrit (HCT) (sensibility: 0.1%), red blood cell distribution width (RDW) (sensibility: 0.1%), and plateletcrit (PCT) (sensibility: 0.001%); the content of hemoglobin (HGB) (sensibility: 1 g/L); mean corpuscular volume (MCV) (sensibility: 0.1 fl), mean corpuscular hemoglobin (MCH) (sensibility: 0.1 pg), mean corpuscular hemoglobin concentration (MCHC) (sensibility: 1 g/L), and mean platelet volume (MPV) (sensibility: 0.1 fl); and platelet distribution width (PDW) (sensibility: 0.1 CV%).

### Blocking ELISA and Quantitation of Cytokines

The quantification of 10 bovine cytokines (GM-CSF, IFN-γ, IL-1b, IL-2, IL-4, IL-5, IL-6, IL-10, IL-12p70, and IL-13) were performed using a commercial cytokine array (QAB-CYT-1) (RayBiotech, Norcross, USA) following the manufacturer's instructions, with sensibilities of 1 × 10^−2^-1 × 10^−5^ pg/ml. Briefly, 100 μl of plasma from each cow was used, with the cytokine standard mix and sample diluent as positive and negative controls, respectively. After blocking, the slides were incubated with 100 μl of samples, 80 μl of biotinylated antibody cocktail, and 80 μl of Cy3 equivalent dye–streptavidin, respectively, and washed thoroughly between each step of incubation. Water droplets were removed completely before the signals were visualized through an Axon GenePix (Molecular Devices, San Jose, USA) with a Cy3 wavelength (green channel).

### Milk Yield, SCS, and Milk Composition

The milk yield of each cow was automatically recorded by milk sensors (Afimilk, Kibbutz, Israel) three times (6.30 a.m., 2.00 and 10.00 p.m.) a day. The 20-ml milk samples collected with 0.015 g of potassium dichromate on the second day of every month were sent to Henan (Zhengzhou DHI center, Henan, China) or Nanjing (Nanjing DHI center, Jiangsu, China) DHI test center to determine the somatic cell count (sensibility: 10 thousand cells/ml) using a Fossomatic™ FC system (FOSS, Hillerod, Denmark), as well as fat (%) (performance range: 2–15%), protein (%) (performance range: 2–10%), and lactose (%) (performance range: 2–10%) using a MilkoScan™ FT+ system (FOSS, Hillerod, Denmark). Further analyses were performed with the SCS calculated as SCC [SCS = log_2_(SCC/100) + 3], as described previously (Yang et al., 2016a).

### Statistical Analysis

All statistical analyses were performed using the STATISTICA 7.0 software package (StatSoft, Oklahoma, USA). Independent Student's *t*-test was used to compare 18 CBC parameters, the expression levels of 10 cytokines, the milk yield (305-day milk yield and average daily milk yield), SCS, and milk composition (fat, protein, and lactose) between the intervention and control groups. The 305-day milk yield of each cow was adjusted and calculated according to the criteria developed by the Dairy Association of China (Supplementary Table S1). In addition, further analyses were performed to determine the effect of BLV infection on milk yield, SCS, and composition between the intervention and control groups in each lactation number (2, 3, and 4) stage (early, middle, and late). The reproduction performance and the incidences of six common diseases between the intervention and control groups were compared using the chi-square test. All data were presented as mean ± SD and considered statistically significant when *P* ≤ 0.050.

## Results

### The Situation of Cows

At the start of this study, 30 first-lactation cows were equally divided into the intervention and control groups, respectively. However, due to some unexpected issues and/or accidents, four cows were dead from heat stress, and 14 cows were culled from herds due to several incurable diseases, while none of the cows in the intervention group have shown one of the most common signs of BLV infection (Table 1). The culling decisions were made independently by the veterinarians. In detail, nine cows in the intervention group were dead (heat stress, *n* = 3) or culled (severe diarrhea, *n* = 2; clinical ketosis, *n* = 1; reproductive syndrome, *n* = 1; severe pneumonia, *n* = 1; dermatitis verrucosa, *n* = 1) between 15 July 2017 and 15 August 2019. Totally 10 cows in the control group were dead (heat stress, *n* = 1) or culled (severe diarrhea, *n* = 1; clinical ketosis, *n* = 2; incurable clinical mastitis, *n* = 1; reproductive syndrome, *n* = 2; severe pneumonia, *n* = 1; dermatitis verrucosa, *n* = 2) between 27 August 2017 and 28 November 2020.

On 07 July 2021, all the remaining cows had finished their fourth lactation. However, at that time, there were only seven and five cows remaining in the herds, respectively. It was predicted that some of these cows will be culled during the following lactation, which will significantly further reduce the power of statistical analysis. Therefore, we decided to end this study and evaluate the effects of BLV infection on cows in lactation numbers 2–4.

### Comparison of Dairy Performance Between the Two Groups Before Artificial Inoculation

In order to make the employed cows in the intervention and control groups comparable, we comprehensively analyzed the parity, age, lactation number, weight, and the available records of daily milk yield (*n* = 8,663), SCS (*n* = 185), and composition [fat (*n* = 140), protein (*n* = 134), and lactose (*n* = 133)] of each cow. Statistical analysis showed that there were no significant differences between the intervention and control groups in parity (all in parity-1), lactation stage (all in their first lactation), age (43.00 ± 1.20 vs. 43.53 ± 1.13, month, *P* = 0.219), weight (345.00 ± 10.81 vs. 345.80 ± 10.63, kg, *P* = 0.840), milk yield (19.74 ± 5.98 vs. 19.60 ± 5.86, kg/d, *P* = 0.254), SCS (1.75 ± 1.85 vs. 2.12 ± 1.79, *P* = 0.180), fat (4.09 ± 0.84 vs. 4.08 ± 0.89, %, *P* = 0.940), protein (3.53 ± 0.46 vs. 3.47 ± 0.58, %, *P* = 0.506), and lactose (5.04 ± 0.18 vs. 5.05 ± 0.21, %, *P* = 0.760) before artificial inoculation (Table 1), indicating that these 30 cows employed in this study were comparable with similar dairy performance.

### Detection BLV Provirus and Gp51 Antibodies by FRET-QPCR and ELISA

**Figure 1:**
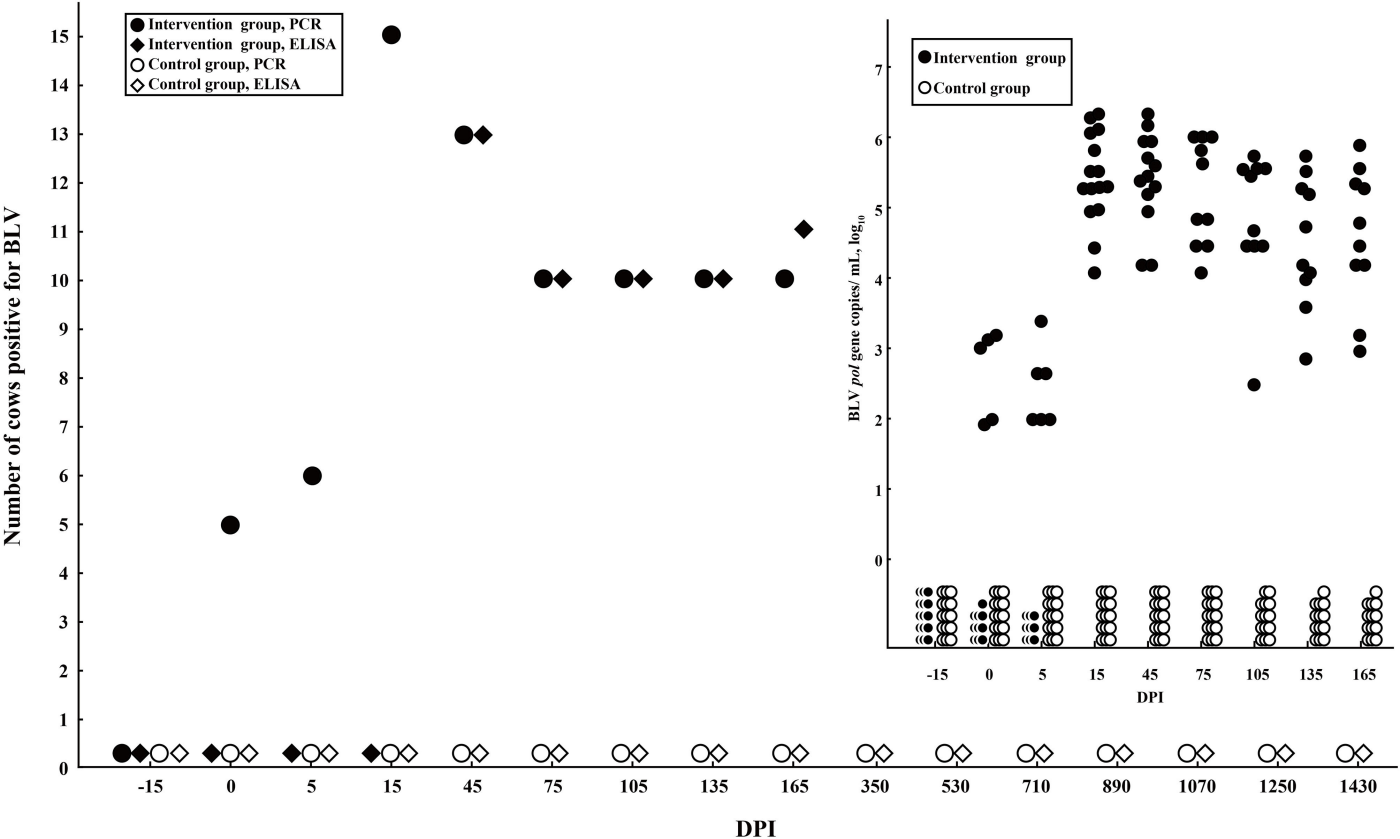
Detection of BLV proviral DNA and anti-BLV antibodies. (Main) BLV FRET-qPCR showed that five and six inoculated cows were detected to be positive at 0 and 5 DPI, respectively. From 45 DPI, all cows in the intervention group were BLV-positive by FRET-qPCR and ELISA. In comparison, all the control cows were free of BLV throughout this study. (Insertion) The copy numbers of BLV *pol* gene of cows in the intervention and control groups were quantified by FRET-qPCR.

To evaluate the viral infection, both FRET-qPCR and commercial blocking ELISA were applied to screen BLV proviral DNA and anti-BLV antibodies. The results showed that BLV proviral DNA was available to be detected in six cows at 0 (2 h) DPI and all the cows at 15 DPI, while anti-BLV gp51 antibodies were unavailable to be detected in any cow during this period. From 45 DPI, BLV proviral DNA and anti-gp51 antibodies could be detected in all the 15 cows in the intervention group, while the control animals remained negative throughout this experiment (Figure 1).

### Complete Blood Count

**Figure 2:**
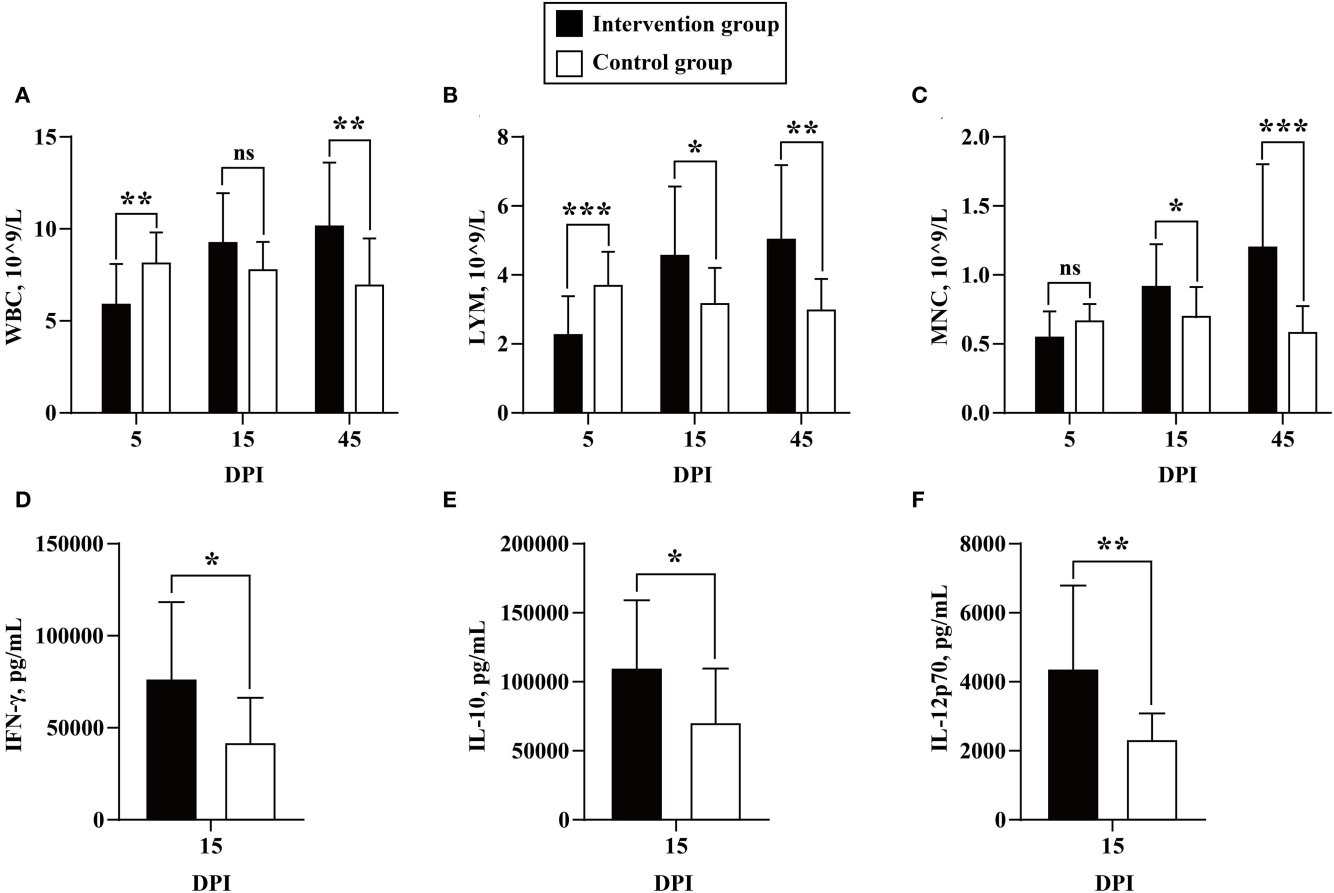
Quantification of CBC parameters and cytokines. The numbers of white blood cells (WBCs) **(A)**, lymphocytes (LYM) **(B)**, and monocytes (MNCs) **(C)** were compared between cows in the intervention (in black) and control (in white) groups at 5, 15, and 45 DPI. The expression levels of IFN-γ **(D)**, IL-10 **(E)**, and IL-12p70 **(F)** were compared between the cows in the inoculation (in black) and control (in black) groups at 15 DPI. Data are shown as mean ± SD, and the symbols indicate *P* ≤ 0.001 (***), *P* ≤ 0.010 (**), *P* ≤ 0.050 (*), and *P* > 0.050 (ns), respectively.

Between 18 May 2017 and 17 November 2017, CBC tests were performed on 241 whole blood samples collected at 15 days before inoculation and at 0, 5, 15, 45, 75, 105, 135, and 165 DPI, including 113 and 128 samples in the intervention and control groups, respectively. Before BLV inoculation, the individuals in the intervention and control groups showed similar and normal CBC parameters compared with reference values (Supplementary Figure S1, Supplementary Tables S2, S3). Our results reliably revealed that the number of WBCs, LYMs, and MNCs of the BLV-inoculated cows decreased immediately after challenge (5 DPI) and subsequently drastically increased (15 and 45 DPI). In detail, at 5 DPI, the number of WBCs, LYMs, and MNCs all decreased in the challenged cows, among which WBCs (5.89 ± 2.20 vs. 8.15 ± 1.65, 10^9^/L, *P* = 0.004) and LYMs (2.27 ± 1.11 vs. 3.67 ± 1.00, 10^9^/L, *P* = 0.001) showed statistical differences. At 15 DPI, there were significant higher levels of LYMs (4.56 ± 2.00 vs. 3.13 ± 1.07, 10^9^/L, *P* = 0.022) and MNCs (0.91 ± 0.32 vs. 0.67 ± 0.23, 10^9^/L, *P* = 0.036) in the challenged cows. At 45 DPI, significant higher numbers of WBCs (10.13 ± 3.48 vs. 6.98 ± 2.50, 10^9^/L, *P* = 0.010), LYMs (5.05 ± 2.13 vs. 2.99 ± 0.90, 10^9^/L, *P* = 0.002), and MNCs (1.19 ± 0.61 vs. 0.58 ± 0.19, 10^9^/L, *P* = 0.001) were observed in the challenged cows (Figures 2A–C, Supplementary Tables S2, S3).

### Quantitation of Cytokines

Between 18 May 2017 and 17 November 2017, the quantitation of 10 bovine cytokines was performed on 128 plasma samples collected before inoculation and at 0, 15, 75, and 165 DPI, including 61 and 67 samples in the intervention and control groups, respectively. The results showed that the expression levels of IFN-γ (75,794.73 ± 42,529.12 vs. 41,065.30 ± 25,216.67, pg/ml, *P* = 0.013), IL-10 (108,555.82 ± 50,554.92 vs. 69,994.84 ± 39,582.16, pg/ml, *P* = 0.031), and IL-12p70 (4,304.16 ± 2,483.99 vs. 2,306.02 ± 779.66, pg/ml, *P* = 0.008) increased significantly in the challenged cows compared with the controls at 15 DPI (Figures 2D–F, Supplementary Tables S4, S5).

### Comparison of Milk Yield, SCS, and Composition Between the Intervention and Control Groups After Artificial Inoculation

Statistical analysis showed that no significant differences were noted between the two groups for the duration in the herds (827.00 ± 697.98 vs. 865.93 ± 596.59, day, *P* = 0.871). From 26 June 2016 to 07 July 2021, a total of 23,468 records of individual daily milk yield were automatically recorded by milk sensors (Afimilk, Kibbutz, Israel). Subsequently, these records archived after artificial inoculation (*n* = 14,805) were divided into nine subsets based on lactation number and stage, including subset 1 (lactation number 2, first-lactation stage, *n* = 2,337), subset 2 (lactation number 2, middle lactation stage, *n* = 2,207), subset 3 (lactation number 2, late lactation stage, *n* = 2,296), subset 4 (lactation number 3, first-lactation stage, *n* = 1,430), subset 5 (lactation number 3, middle lactation stage, *n* = 1,428), subset 6 (lactation number 3, late lactation stage, *n* = 1,432), subset 7 (lactation number 4, first-lactation stage, *n* = 1,220), subset 8 (lactation number 4, middle lactation stage, *n* = 1,216), and subset 9 (lactation number 4, first-lactation stage, *n* = 1,239). At the same time, a total of 1,742 of records of milk SCS and composition were obtained from monthly performed DHI tests, including 492 of milk SCS (lactation numbers 1/2/3/4: 185/133/93/81), 451 of milk fat (lactation numbers 1/2/3/4: 140/141/89/81), 440 of milk protein (lactation numbers 1/2/3/4: 134/137/95/74), and 359 of milk lactose (lactation numbers 1/2/3: 133/143/83), respectively. Then these records were divided into different subsets according to the same criteria.

**Figure 3:**
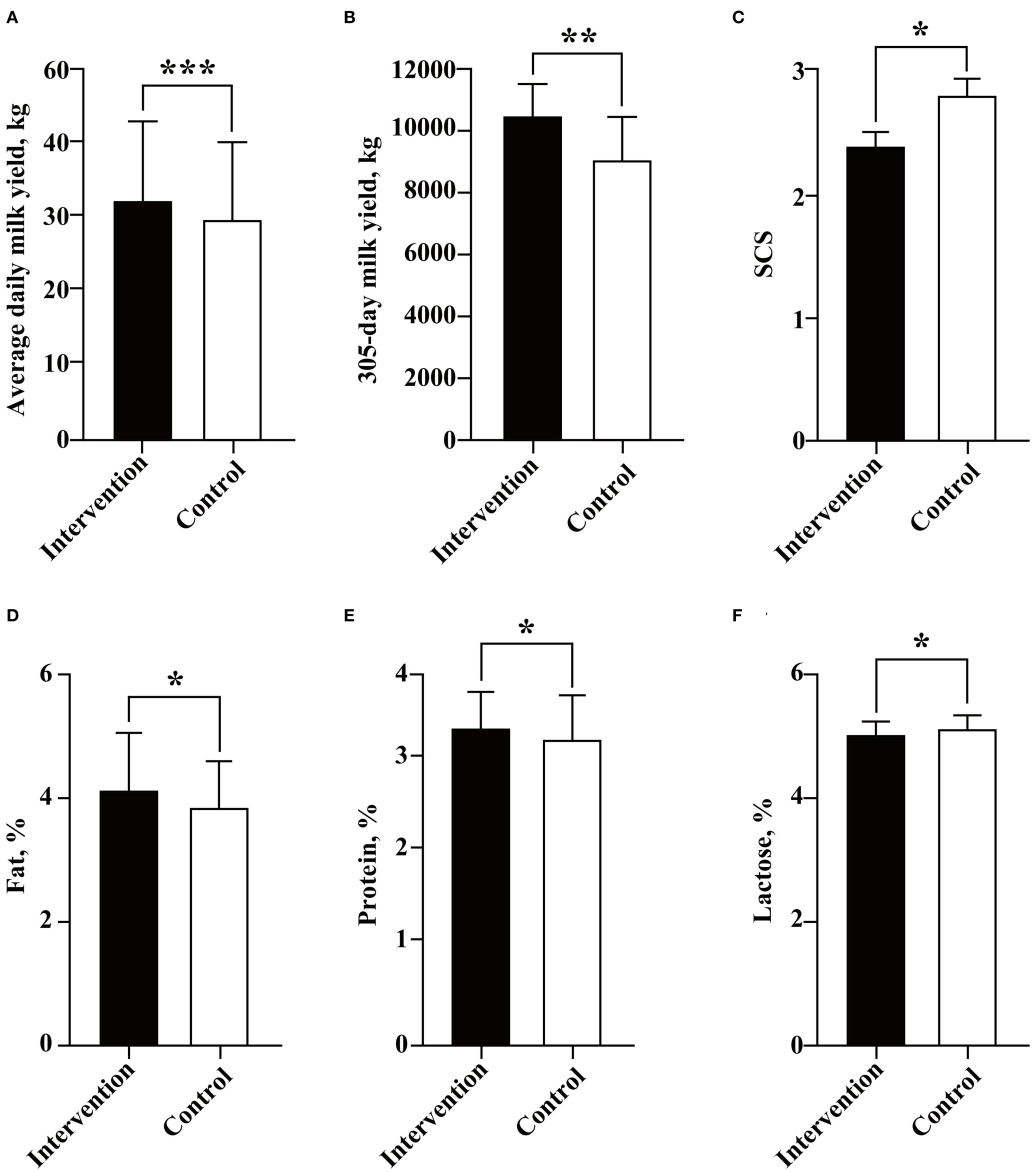
Association between BLV infection and dairy performance of cows. The average daily milk yield **(A)**, 305-day milk yield **(B)**, SCS **(C)**, and contents of fat **(D)**, protein **(E)**, and lactose **(F)** were compared and analyzed between the intervention (in black) and control (in white) groups by Student's *t*-test. Data are shown as mean ± SD, and the symbols indicate *P* ≤ 0.001 (***), *P* ≤ 0.010 (**), and *P* ≤ 0.05 (*), respectively.

**Figure 4:**
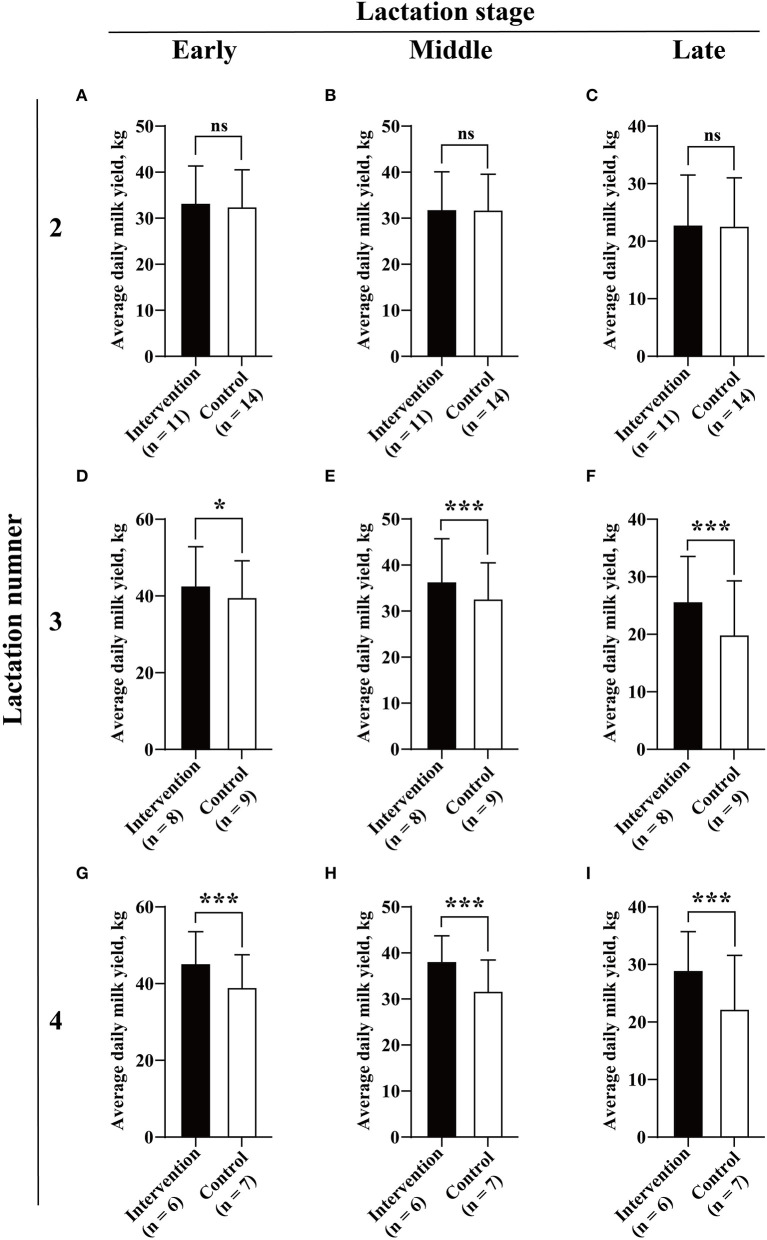
Association between BLV infection and milk yield. Average daily milk yield **(A–I)** for each of three lactation numbers (2, 3, and 4) and stages (early, middle, and late) were compared between the intervention (in black) and control (in white) groups by Student's *t*-test. Data are shown as mean ± SD, and the symbols indicate *P* ≤ 0.001 (***), *P* ≤ 0.050 (*), and *P* > 0.050 (ns), respectively.

When we compared the overall milk yield between the two groups, independent Student's *t*-test was used to compare the average daily milk yield and 305-day milk yield between the two groups. The results showed that the cows in the intervention group had significant increased average daily milk production (32.53 ± 10.79 vs. 29.74 ± 10.77, kg/d, *P* < 0.001) and adjusted 305-day milk yield (10,550.07 ± 1,127.20 vs. 8,979.51 ± 1,541.15, kg, *P* = 0.002) than the uninfected cows (Figures 3A,B). Subsequently, further analysis was carried out to determine the association between BLV infection and milk yield in cows in different lactation numbers (2, 3, and 4) and stages (first, middle, and late). Interestingly, from the early lactation stage of lactation number 3 to the end of this study, the infection of BLV was determined to be associated with significantly increased milk yield. In detail, the average daily milk yield of cows in the intervention group was significantly higher than that of the control at all lactation stages of lactation number 3 (seven intervened cows and nine or seven control cows) (early: 40.68 ± 12.33 vs. 39.32 ± 9.88, kg/d, *P* = 0.021; middle: 36.34 ± 9.43 vs. 32.28 ± 8.19, kg/d, *P* < 0.001; late: 25.26 ± 8.49 vs. 19.69 ± 9.59, kg/d, *P* < 0.001) and lactation number 4 (six intervened cows and seven control cows) (44.90 ± 9.86 vs. 38.62 ± 8.92, kg/d, *P* < 0.001; middle: 37.58 ± 6.41 vs. 31.27 ± 7.09, kg/d, *P* < 0.001; late: 29.12 ± 6.36 vs. 22.07 ± 9.51, kg/d, *P* < 0.001) (Figure 4).

**Figure 5:**
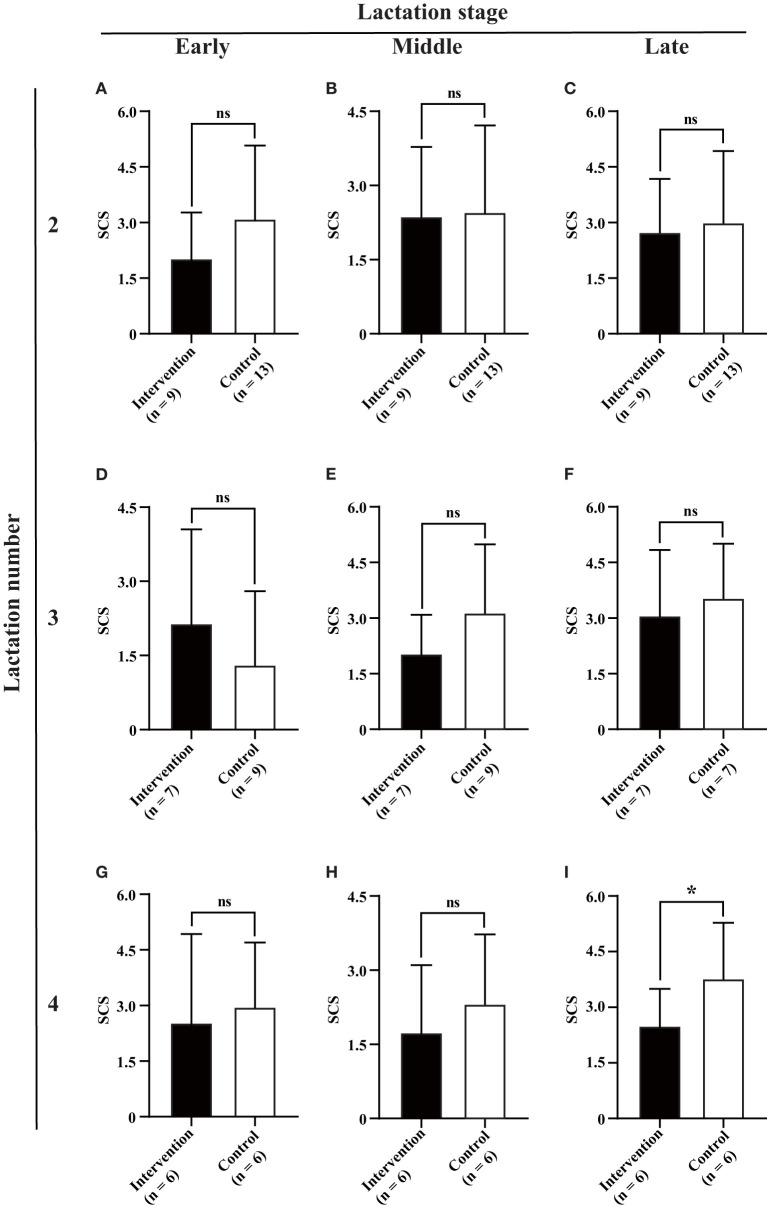
Association between BLV infection and milk SCS. Average monthly milk SCS **(A–I)** for each of three lactation numbers (2, 3, and 4) and stages (early, middle, and late) was compared between the intervention (in black) and control (in white) groups by Student's *t*-test. Data are shown as mean ± SD, and the symbols indicate *P* ≤ 0.050 (*) and *P* > 0.050 (ns), respectively.

**Figure 6:**
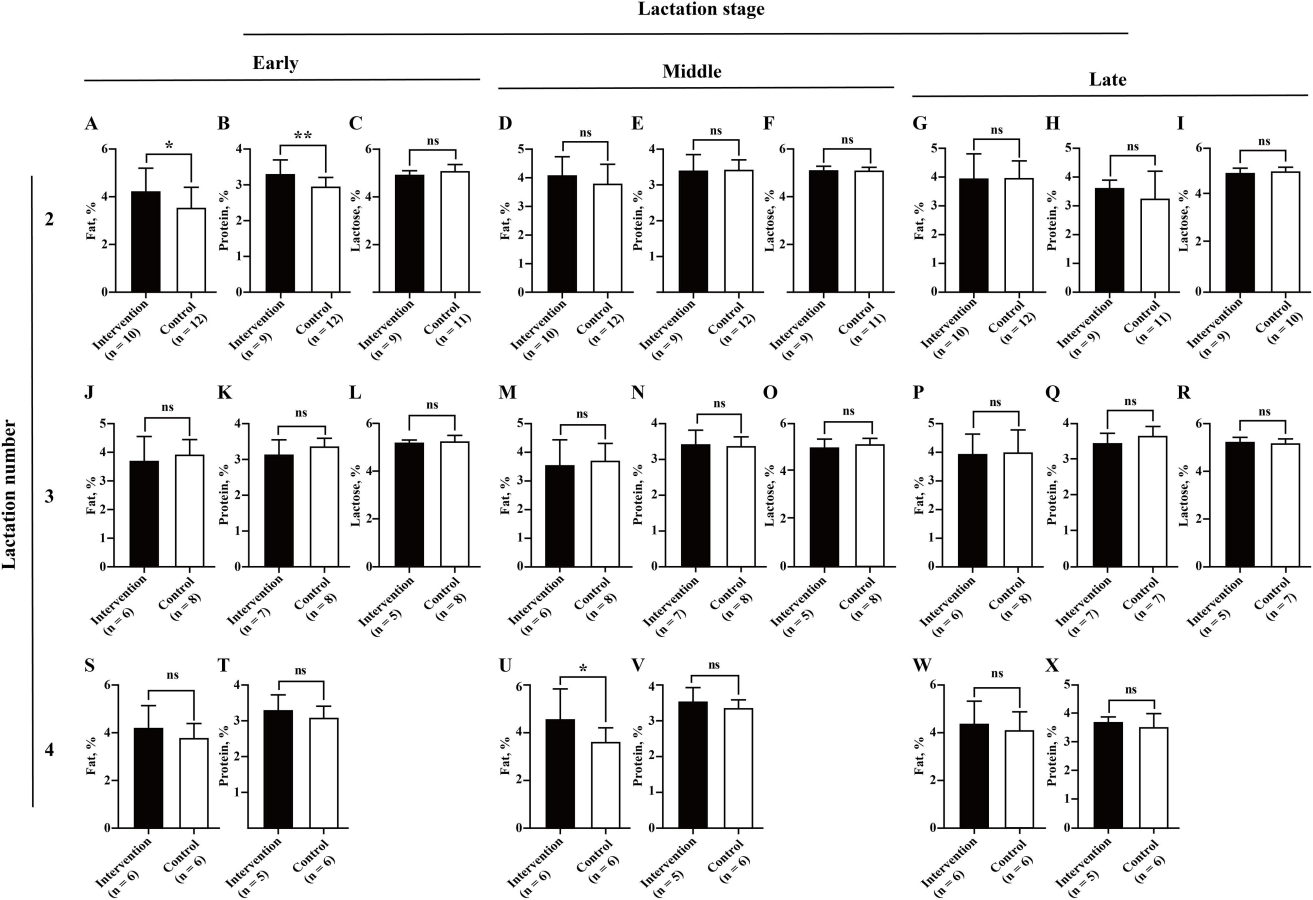
Association between BLV infection and milk composition (fat, protein, and lactose). Average monthly milk fat **(A,D,G,J,M,P,S,U,W)**, protein **(B,E,H,K,N,Q,T,V,X)**, and lactose **(C,F,I,L,O,R)** for each of three lactation numbers (2, 3, and 4) and stages (early, middle, and late) were compared between the intervention (in black) and control (in white) groups by Student's *t*-test. Data are shown as mean ± SD, and the symbols indicate *P* ≤ 0.050 (*) and *P* > 0.050 (ns), respectively.

Although the infection of BLV was determined to be associated with reduced milk SCS (2.40 ± 1.56 vs. 2.81 ± 1.87, *P* = 0.038) and lactose (5.04 ± 0.24 vs. 5.11 ± 0.24, %, *P* = 0.036), and increased milk fat (4.00 ± 0.98 vs. 3.80 ± 0.71, %, *P* = 0.031) and protein (3.40 ± 0.41 vs. 3.30 ± 0.51, %, *P* = 0.050) (Figures 3C–F), statistical differences were only observed at the late lactation stage of lactation number 4 (six intervened cows and six control cows) (2.55 ± 0.93 vs. 3.73 ± 1.58, *P* = 0.036) for milk SCS (Figure 5), the early lactation stage of lactation number 2 (10 intervened cows and 12 control cows) (4.16 ± 1.00 vs. 3.50 ± 0.86, %, *P* = 0.021), and the middle lactation stage of lactation number 4 (six intervened cows and six control cows) (4.51 ± 1.39 vs. 3.58 ± 0.63, %, *P* = 0.025) for milk fat and early lactation stage of lactation number 2 (nine intervened cows and 12 control cows) (3.26 ± 0.44 vs. 2.93 ± 0.27, %, *P* = 0.005) for milk protein (Figure 6).

### Comparison of Key Performance Indicators (KPIs) Between the Intervention and Control Groups

To investigate in depth other factors that could have affected the dairy performance of cow uncertainty, four universally used KPIs in reproduction (days at first breeding, the percentage of pregnant cows at 150 DIM, and the frequencies of pregnancy loss <90 or ≥90 days of pregnancy) and the incidences of six common diseases (the frequencies of ketosis, metritis, retained placenta, clinical mastitis, lameness, and enteritis) were compared between cows in the intervention and control groups. The data of six common diseases were only available for the cows in lactation numbers 3–4, due to a breakdown on the data storage system. The results showed that in lactation numbers 1–4, cows in the intervention and control groups had similar reproduction performance, including days at first breeding, the rate of pregnancy at 150 DIM, and the rate of pregnancy loss <90 or ≥90 days of pregnancy (*P* > 0.05). Similarly, no significant differences were observed in the incidences of ketosis, metritis, retained placenta, clinical mastitis, lameness, and enteritis between the two groups in lactation numbers 3–4 (*P* > 0.05) (Table 2).

**Table 2:** Comparison of four key performance indicators and the incidences of six common diseases between the intervention and control groups.

**Key performance indicators**	**Lactation number**	**Intervention group**	**Control group**	***P*** **-value (χ^2^)**
Days at first breeding, d		389–408	385–405	
Pregnant cows at 150 DIM, %	1	86.67% (13/15)	86.67% (12/15)	>0.05
	2	77.77% (7/9)	76.92% (10/13)	>0.05
	3	57.14% (4/7)	55.56% (5/9)	>0.05
	4	66.67% (4/6)	66.67% (4/6)	>0.05
	Total	75.67% (28/37)	72.09% (31/43)	>0.05
Pregnancy loss <90 days of pregnancy, %	1–2	0% (0/24)	0% (0/28)	>0.05
	3	14.29% (1/7)	22.22% (2/9)	>0.05
	4	16.67% (1/6)	16.67% (1/6)	>0.05
	Total	5.41% (2/37)	6.98% (3/43)	>0.05
Pregnancy loss > 90 days of pregnancy,%	1–2, 4	0% (0/30)	0% (0/34)	>0.05
	3	0% (0/7)	11.11% (1/9)	>0.05
	Total	0% (0/37)	2.33% (1/43)	>0.05
Ketosis, %	1–2	NA^a^	NA	
	3	14.29% (1/7)	0% (0/9)	>0.05
	4	66.67% (4/6)	66.67% (4/6)	>0.05
	Total	38.46% (5/13)	26.67% (4/15)	>0.05
Metritis, %	1–2	NA	NA	
	3	0% (0/7)	0% (0/9)	>0.05
	4	50.00% (3/6)	83.33% (5/6)	>0.05
	Total	23.08% (3/13)	20.00% (5/15)	>0.05
Retained placenta, %	1–2	NA	NA	
	3	0% (0/7)	11.11% (1/9)	>0.05
	4	16.67% (1/6)	0% (0/6)	>0.05
	Total	7.69% (1/13)	6.67% (1/15)	>0.05
Clinical mastitis, %	1–2	NA	NA	
	3	42.86% (3/7)	33.33% (3/9)	>0.05
	4	33.33% (2/6)	0% (0/6)	>0.05
	Total	38.46% (5/13)	20.00% (3/15)	>0.05
Lameness, %	1–2	NA	NA	
	3	28.57% (2/7)	11.11% (1/9)	>0.05
	4	16.67% (1/6)	50.00% (3/6)	>0.05
	Total	23.08% (3/13)	26.67% (4/15)	>0.05
Enteritis, %	1–2	NA	NA	
	3	28.57% (2/7)	11.11% (1/9)	>0.05
	4	16.67% (1/6)	33.33% (2/6)	>0.05
	Total	23.08% (3/13)	20.00% (3/15)	>0.05

a *Data on the incidence of ketosis, metritis, retained placenta, clinical mastitis, lameness, and enteritis were not available for cows in lactation numbers 1–2*.

## Discussion

It is prohibitively expensive to design and conduct a parallel-group trial with BLV infection with sufficient statistical power. Hutchinson determined the timing and described early fluctuations of BLV detection by qPCR, ELISA, and lymphocyte counts following experimental BLV inoculation with 23 Holstein steers (15 infected steers and eight control steers) (Hutchinson et al., 2020). Benitez evaluated the potential for BLV transmission during natural breeding between a BLV-infected bull and 40 uninfected heifers (20 control heifers and 20 challenged heifers) (Benitez et al., 2019). However, to the best of our knowledge, no study so far has investigated the effects of BLV infection on dairy performance based on artificially inoculated cows. To establish a BLV challenge model with comparable cows, from a BLV-free farm with 2,988 cows, 30 healthy first-lactation cows with similar performance were employed. All these cows were in their second pregnancy with the similar dairy performance in the first lactation. According to the previous large-scale molecular epidemiological studies, BLV genotype 6 was demonstrated to be the endemic strain in China, which shared a significant nucleotide/amino acid polymorphism with FLK-BLV (genotype 1) (Yang et al., 2019a,b; Yu et al., 2019). Hence, in this study, the artificial inoculation was performed with PBMC from a Holstein cow infected with high PVL of BLV genotype 6.

The detection of BLV proviral DNA and gp51 antibody showed that the BLV challenge model was successfully established, and no cross-infection occurred from the beginning to the end of the experiment. For convenience, the hematological diagnostic method based on LYM counts was widely used as the screening and disease severity monitoring tool for BLV, especially among greater parity animals (Nieto Farias et al., 2018; Wisnieski et al., 2020). In this study, we indicated that the most significant increases in WBC, LYM, and MNC counts were all observed at 45 DPI, but not at the later stage of infection. Therefore, it is strongly recommended to use real-time PCR or ELISA, instead of complete blood count for the detection of BLV infection.

The antibody-based detection tests (agar gel immunodiffusion and ELISA) for the diagnosis of BLV infection have been authorized by the World Organisation for Animal Health OIE, 2018. Klintevall et al. (1994) reported that BLV antibodies could be detected by ELISA on 26 DPI. Evermann et al. found that the experimental calves seroconverted to BLV within 8 and 14 weeks, respectively, depending on the equivalent of inoculation (Evermann et al., 1986). Moreover, it is convenient to screen for BLV infection using milk from the DHI sampling process (Nekouei et al., 2015b). In this study, the anti-gp51 antibody was available to be detected since 45 DPI, which emphasized the early diagnostic importance of real-time qPCR.

A retrospective study indicated that the mRNA expression levels of IFN-γ and IL-12 were significantly higher in PBMC from infected cattle with low and high PVL than uninfected animals (Farias et al., 2016). In this study, the significant higher expression levels of IFN-γ and IL-12p70 in plasma were observed during the early stage of BLV infection (15 DPI). In addition, the expression level of IL-10 was also significant higher in plasma of infected individuals.

The main purpose of this study was to better understand the possible association between BLV infection and the dairy performance of cows. Although several previous studies have provided evidence that BLV infection is associated with decreased milk yield at herd or individual levels (Erskine et al., 2012; Nekouei et al., 2016; Norby et al., 2016), other studies indicated that the BLV infected rate is not associated with milk production (Sorge et al., 2011). Some studies have even shown that the herd with a higher BLV-positive rate actually produce more milk (Abdalla et al., 2016). It has been well revealed that individual dairy performance of cows can be greatly influenced by a variety of factors, such as breed, parity, lactation stage, feeding conditions, and the occurrence of diseases (Inchaisri et al., 2010; Soufleri et al., 2019; Barth, 2020; Fehlberg et al., 2020; Goncalves et al., 2020; Adriaens et al., 2021). Therefore, to minimize these effects, the 30 cows enrolled in this study were comparable with the identical parity/lactation number, similar age, dairy performance, and predicted calving date before BLV inoculation. This is one of the advantages of this experiment over other observational studies. In addition, to create greater certainty in our conclusion, milk yield, SCS, and milk composition (fat, protein, and lactose) were analyzed in the intervention and control groups according to different lactation numbers (2, 3, and 4) and stages (early, middle, and late). Unexpectedly, our 4-year study showed that BLV-infected cows had significant increased milk yield compared with the uninfected cows. At the same time, there were statistical differences in milk yield between the two groups from the early lactation stage of lactation number 3 to the end of this study. Although the data of six common diseases were only available for the cows in lactation numbers 3–4, the available data indicated that differences in reproduction performance and the incidence of diseases would not contribute to the significant increased milk yield in the intervention group in lactation numbers 3–4. The Canadian and U.S. studies determined a negative association between herd-level milk production and BLV positivity (Sargeant et al., 1997; Erskine et al., 2012), based on AGID and ELISA, respectively. A recent study indicated that BLV-infected cows with two and three lactations showed significantly lower life milk productions than their negative counterparts (Nekouei et al., 2016). These results are at odds with this study, but we did not analyze the lifetime milk yield. However, our results are partially consistent with a previous study in 16 U.S. states, which indicated that selection for higher milk yield may lead to increased BLV prevalence in dairy herds (Abdalla et al., 2016). By contrast, although the overall milk SCS and composition varied between the intervention and control groups, there were almost no significant differences in the lactation number and stage-based analysis, which may be due to the limited sample size. However, it should be noted that in previous case–control studies, it is not really clear which genotype(s) of BLV had infected those enrolled cows, probably BLV genotype 1, the most widespread genotype of BLV in the world. This variability could be a source of differences in results among different studies. Our study has some inherent limitations that could have biased our results. First, the limited number of experimental animals may affect the reliability of the results. Second, the individual differences and lack of specific pathogen-free rearing conditions will interfere with the control of variables. Third, although the farm managers and workers were supposed to be blinded as to which herd of cows was challenged, they frequently figured it out. Therefore, more similar studies were recommended to be conducted to clarify and mitigate such detrimental effects.

## Data Availability Statement

The original contributions presented in the study are included in the article/Supplementary Material, further inquiries can be directed to the corresponding author/s.

## Ethics Statement

The animal study was reviewed and approved by the Institutional Animal Care and Use Committee of the Yangzhou University College of Veterinary Medicine, China.

## Author Contributions

YY, CW, and ZY contributed to conception and design of the study. YY, ZG, YL, JZ, YP, SC, WC, and HW performed the experiments. YY, ZG, XL, YM, XH, JS, and AQ organized the database. YY, ZG, XL, and YM performed the statistical analysis. YY wrote the first draft of the manuscript. CW, SS, and ZY revised the manuscript. All authors contributed to the article and approved the submitted version.

## Funding

This work was funded by the National Natural Science Foundation of China (32002263 to YY), Basic Research Program of Jiangsu Province (BK20190881 to YY), Postdoctoral Research Foundation of China (2019M650126 to YY), Natural Science Foundation of Jiangsu Higher Education Institutions of China (19KJB230001 to YY), Seed Industry Vitalization Program of Jiangsu Province (JBGS[2021]117 to YY), Jiangsu Agricultural Science and Technology Independent Innovation Fund (CX(20)3089 to YY), and Priority Academic Program Development of Jiangsu Higher Education Institutions (NA to CW, SS, and ZY). The funders had no role in study design, data collection and analysis, decision to publish, or preparation of the manuscript.

## Conflict of Interest

The authors declare that the research was conducted in the absence of any commercial or financial relationships that could be construed as a potential conflict of interest.

## Publisher's Note

All claims expressed in this article are solely those of the authors and do not necessarily represent those of their affiliated organizations, or those of the publisher, the editors and the reviewers. Any product that may be evaluated in this article, or claim that may be made by its manufacturer, is not guaranteed or endorsed by the publisher.
