# Supplementary material for: Dairy Cows Experimentally Infected With Bovine Leukemia Virus Showed an Increased Milk Production in Lactation Numbers 3–4: A 4-Year Longitudinal Study
Source: Front Microbiol. 2022 Jul 11;13:946463. doi: 10.3389/fmicb.2022.946463 (PMC9309534; doi:10.3389/fmicb.2022.946463)
Supplement: Supplementary file 1 [file Data_Sheet_1.docx]

Supplementary Material

Cows experimentally infected with bovine leukemia virus showed an increased milk production: a four-year longitudinal study

Yi Yang ^1,2*^, Zaicheng Gong ^1^, Yi Lu ^1^, Xubin Lu ^3^, Jilei Zhang ^4^, Ye Meng ^1^, Yalan Peng ^1^, Shuangfeng Chu ^3^, Wenqiang Cao ^1^, Xiaoli Hao ^1^, Jie Sun ^5^, Heng Wang ^1^, Aijian Qin ^1,2^, Chengming Wang ^6^, Shaobin Shang ^1,2*^, Zhangping Yang ^3*^

^1^Jiangsu Co-innovation Center for Prevention and Control of Important Animal Infectious Diseases and Zoonoses; College of Veterinary Medicine, Yangzhou University, Yangzhou 225009, Jiangsu, China

^2^International Corporation Laboratory of Agriculture and Agricultural Products Safety, Yangzhou University, Yangzhou 225009, Jiangsu, China

^3^College of Animal Science and Technology, Yangzhou University, Yangzhou 225009, Jiangsu, China

^4^Division of Gastroenterology and Hepatology, Department of Medicine, University of Illinois at Chicago, Chicago 60607, Illinois, United States of America

^5^Shenzhen Academy of Inspection and Quarantine Sciences, Shenzhen 518045, Guangdong, China

^6^Department of Pathobiology, College of Veterinary Medicine, Auburn University, Auburn 36849, Alabama, United States of America

*** Correspondence:**yangyi@yzu.edu.cn (YY), shaobinshang@yzu.edu.cn (SS) and yzp@yzu.edu.cn (ZY)

**Supplementary Table S1.** The correction coefficients for the adjustment and calculation of 305-day milk yield.

| **Parity** | **Days in milk** | **240** | **250** | **260** | **270** | **280** | **290** | **300** | **305** | **310** | **320** | **330** | **340** | **350** | **360** | **370** |
| --- | --- | --- | --- | --- | --- | --- | --- | --- | --- | --- | --- | --- | --- | --- | --- | --- |
| **1** |  | 1.182^1^ | 1.148 | 1.116 | 1.036 | 1.055 | 1.031 | 1.011 | 1.000 | 0.987 | 0.965 | 0.947 | 0.924 | 0.911 | 0.895 | 0.881 |
| **2 - 5** |  | 1.165 | 1.133 | 1.103 | 1.077 | 1.052 | 1.031 | 1.011 | 1.000 | 0.988 | 0.970 | 0.952 | 0.936 | 0.925 | 0.911 | 0.904 |
| **> 6** |  | 1.155 | 1.123 | 1.094 | 1.070 | 1.047 | 1.025 | 1.009 | 1.000 | 0.988 | 0.970 | 0.956 | 0.900 | 0.928 | 0.916 | 0.993 |

^1^These correction coefficients were referred to the criteria developed by Dairy Association of China.

**Supplementary Table S2.** The detection of 18 CBC parameters in the intervention and control groups at -15, 0, 5, 15 and 45 DPI.

| **DPI** | **-15** | | **0** | | **5** | | **15** | | **45** | |
| --- | --- | --- | --- | --- | --- | --- | --- | --- | --- | --- |
| **CBC** | **Intervention** | **Control** | **Intervention** | **Control** | **Intervention** | **Control** | **Intervention** | **Control** | **Intervention** | **Control** |
| **WBC, 10^9^/L** | 7.41 ± 1.62^1^ | 7.38 ± 1.99 | 7.90 ± 1.41 | 8.35 ± 1.48 | 5.89 ± 2.20 | 8.15 ± 1.65 | 9.27 ± 2.67 | 7.79 ± 1.51 | 10.13 ± 3.48 | 6.98 ± 2.50 |
| **LYM, 10^9^/L** | 3.15 ± 1.17 | 3.39 ± 1.11 | 3.57 ± 0.97 | 3.67 ± 1.02 | 2.27 ± 1.11 | 3.67 ± 1.00 | 4.56 ± 2.00 | 3.13 ± 1.07 | 5.05 ± 2.13 | 2.99 ± 0.90 |
| **LYM%, %** | 41.45 ±10.48 | 46.13 ± 7.64 | 44.97 ± 7.74 | 43.54 ± 7.11 | 37.57 ± 6.45 | 44.30 ± 4.75 | 48.05 ± 12.02 | 39.85 ± 9.87 | 48.95 ± 8.81 | 44.84 ± 10.80 |
| **MNC, 10^9^/L** | 0.63 ± 0.19 | 0.62 ± 0.19 | 0.56 ± 0.16 | 0.72 ± 0.15 | 0.55 ± 0.19 | 0.66 ± 0.13 | 0.91 ± 0.32 | 0.69 ± 0.23 | 1.19 ± 0.61 | 0.58 ± 0.19 |
| **MNC%, %** | 8.67 ± 1.89 | 8.64 ± 1.80 | 7.19 ± 1.58 | 8.69 ± 1.72 | 9.87 ± 2.11 | 8.37 ± 1.48 | 10.00 ± 2.14 | 8.97 ± 2.55 | 11.62 ± 3.14 | 8.47 ± 1.54 |
| **GRAN, 10^9^/L** | 3.64 ± 0.98 | 3.37 ± 1.06 | 3.77 ± 0.81 | 3.96 ± 0.76 | 3.07 ± 1.05 | 3.82 ± 0.71 | 3.81 ± 1.35 | 3.97 ± 0.89 | 3.89 ± 1.15 | 3.41 ± 1.69 |
| **GRAN%, %** | 49.88 ± 10.91 | 45.24 ± 7.64 | 47.85 ± 7.80 | 47.77 ± 6.65 | 52.55 ± 5.98 | 47.33 ± 4.64 | 41.95 ± 12.22 | 51.17 ± 8.57 | 39.43 ± 7.41 | 46.69 ± 11.03 |
| **RBC, 10^12^/L** | 6.32 ± 0.47 | 6.30 ± 0.70 | 6.33 ± 0.47 | 6.39 ± 0.57 | 6.06 ± 0.70 | 6.37 ± 0.60 | 6.16 ± 0.41 | 6.25 ± 0.64 | 5.69 ± 0.61 | 5.72 ± 0.59 |
| **HGB, g/L** | 112.07 ± 10.67 | 109.63 ± 8.97 | 111.87 ± 8.62 | 111.27 ± 7.04 | 105.53 ± 9.42 | 110.07 ± 8.32 | 108.53 ± 7.35 | 109.13 ± 9.42 | 96.62 ± 10.71 | 99.80 ± 9.94 |
| **HCT, %** | 32.17 ± 3.34 | 31.19 ± 2.63 | 31.83 ± 2.68 | 31.37 ± 2.20 | 30.62 ± 3.24 | 31.37 ± 2.56 | 31.19 ± 2.25 | 30.97 ± 2.91 | 28.33 ± 3.54 | 28.97 ± 3.19 |
| **MCV, fL** | 51.02 ± 4.57 | 49.86 ± 3.14 | 50.51 ± 4.43 | 49.37 ± 3.23 | 50.85 ± 4.40 | 49.51 ± 3.32 | 50.82 ± 4.12 | 49.77 ± 3.38 | 50.07 ± 4.93 | 50.81 ± 3.55 |
| **MCH, pg** | 17.69 ± 1.43 | 17.41 ± 1.04 | 17.67 ± 1.37 | 17.43 ± 1.04 | 17.45 ± 1.33 | 17.31 ± 1.06 | 17.62 ± 1.32 | 17.45 ± 1.03 | 17.02 ± 1.49 | 17.44 ± 1.18 |
| **MCHC, g/L** | 348.27 ± 6.77 | 351.00 ± 6.31 | 351.27 ± 7.81 | 354.40 ± 8.66 | 344.87 ± 8.25 | 350.60 ± 6.57 | 347.60 ± 6.73 | 352.07 ± 6.72 | 341.15 ± 9.97 | 344.53 ± 6.71 |
| **RDW, %** | 17.13 ± 1.14 | 17.00 ± 0.65 | 16.76 ± 0.84 | 16.76 ± 0.71 | 16.91 ± 0.95 | 16.77 ± 0.74 | 16.25 ± 0.94 | 16.41 ± 0.69 | 16.14 ± 0.77 | 16.59 ± 0.63 |
| **PLT, 10^9^/L** | 233.07 ± 137.08 | 263.63 ± 126.57 | 363.40 ± 125.66 | 308.33 ± 109.30 | 353.20 ± 71.88 | 364.80 ± 81.92 | 316.00 ± 118.17 | 304.60 ± 151.00 | 388.08 ± 206.35 | 414.53 ±135.16 |
| **MPV, fL** | 5.40 ± 0.30 | 5.56 ± 0.45 | 5.56 ± 0.32 | 5.67 ± 0.39 | 5.51 ± 0.33 | 5.50 ± 0.37 | 5.61 ± 0.61 | 5.63 ± 0.52 | 5.16 ± 0.41 | 5.29 ± 0.39 |
| **PDW, CV%** | 16.05 ± 0.34 | 16.07 ± 0.41 | 15.89 ± 0.36 | 15.92 ± 0.19 | 15.97 ± 0.29 | 16.03 ± 0.37 | 16.05 ± 0.37 | 15.96 ± 0.41 | 15.90 ± 0.41 | 15.87 ± 0.43 |
| **PCT, %** | 0.12 ± 0.07 | 0.14 ± 0.06 | 0.20 ± 0.07 | 0.17 ± 0.06 | 0.19 ± 0.04 | 0.20 ± 0.04 | 0.17 ± 0.06 | 0.17 ± 0.08 | 0.20 ± 0.11 | 0.22 ± 0.06 |

^1^Data were shown as mean ± SD.

**Supplementary Table S3.** The detection of 18 CBC parameters in the intervention and control groups at 75, 105, 135 and 165 DPI.

| **DPI** | **75** | | **105** | | **135** | | **165** | |
| --- | --- | --- | --- | --- | --- | --- | --- | --- |
| **CBC** | **Intervention** | **Control** | **Intervention** | **Control** | **Intervention** | **Control** | **Intervention** | **Control** |
| **WBC, 10^9^/L** | 7.93 ± 2.27^1^ | 6.85 ± 1.90 | 9.09 ± 2.40 | 7.49 ± 1.99 | 9.28 ± 2.82 | 9.00 ± 3.73 | 10.19 ± 3.51 | 8.68 ± 1.99 |
| **LYM, 10^9^/L** | 3.73 ± 1.46 | 2.59 ± 0.85 | 3.65 ±1.32 | 2.75 ± 0.90 | 4.18 ± 2.43 | 2.93 ± 0.66 | 4.84 ± 2.75 | 3.00 ± 1.00 |
| **LYM%, %** | 46.70 ± 10.49 | 38.67 ± 11.35 | 40.20 ± 11.37 | 37.45 ± 9.81 | 42.48 ± 13.22 | 35.16 ± 10.13 | 44.98 ± 11.83 | 34.67 ± 9.63 |
| **MNC, 10^9^/L** | 0.86 ± 0.38 | 0.65 ± 0.22 | 1.03 ± 0.53 | 0.69 ± 0.19 | 0.79 ± 0.30 | 0.77 ± 0.24 | 0.99 ± 0.43 | 0.87 ± 0.21 |
| **MNC%, %** | 10.75 ± 2.15 | 9.82 ± 1.48 | 11.20 ± 2.85 | 9.47 ± 1.09 | 8.58 ± 1.87 | 8.90 ± 1.08 | 10.05 ± 3.41 | 10.12 ± 1.19 |
| **GRAN, 10^9^/L** | 3.34 ± 1.05 | 3.61 ± 1.48 | 4.41 ± 1.44 | 4.04 ± 1.64 | 4.31 ± 0.88 | 5.30 ± 3.24 | 4.36 ± 0.91 | 4.82 ± 1.46 |
| **GRAN%, %** | 42.55 ± 9.67 | 51.51 ± 11.59 | 48.60 ± 10.00 | 53.08 ± 10.06 | 48.94 ± 12.88 | 55.94 ± 10.58 | 44.97 ± 9.90 | 55.20 ± 9.06 |
| **RBC, 10^12^/L** | 5.07 ± 0.36 | 5.70 ± 0.99 | 5.68 ± 0.50 | 6.18 ± 0.82 | 6.13 ± 0.46 | 6.13 ± 0.53 | 6.30 ± 0.44 | 6.10 ± 0.36 |
| **HGB, g/L** | 84.40 ± 6.36 | 97.20 ±17.31 | 91.40 ± 4.67 | 102.07 ± 7.94 | 95.90 ± 6.40 | 100.09 ± 8.37 | 101.00 ± 5.81 | 101.67 ± 7.58 |
| **HCT, %** | 24.01 ± 1.92 | 27.63 ± 5.35 | 25.72 ± 1.47 | 29.15 ± 2.21 | 27.04 ± 2.01 | 28.48 ± 2.43 | 27.78 ± 2.15 | 28.14 ± 2.27 |
| **MCV, fL** | 47.59 ± 4.36 | 48.65 ± 3.70 | 45.63 ± 4.37 | 47.60 ± 3.67 | 44.32 ± 3.61 | 46.70 ± 3.78 | 44.22 ± 2.95 | 46.33 ± 4.07 |
| **MCH, pg** | 16.63 ± 1.30 | 17.06 ± 1.20 | 16.12 ± 1.22 | 16.60 ± 1.34 | 15.64 ± 1.05 | 16.33 ± 1.24 | 16.01 ± 0.99 | 16.65 ± 1.34 |
| **MCHC, g/L** | 351.10 ± 7.52 | 352.13 ± 6.91 | 355.20 ± 11.55 | 349.57 ± 5.06 | 354.40 ± 9.20 | 351.00 ± 5.92 | 363.80 ± 9.32 | 360.92 ± 6.61 |
| **RDW, %** | 16.74 ± 0.62 | 16.20 ± 0.67 | 16.89 ± 0.87 | 16.42 ± 0.55 | 16.66 ± 0.79 | 16.28 ± 0.70 | 17.04 ± 0.85 | 16.53 ± 1.07 |
| **PLT, 10^9^/L** | 500.70 ± 131.80 | 381.53 ± 144.88 | 457.30 ± 63.83 | 432.43 ± 70.29 | 469.20 ± 80.67 | 393.09 ± 146.57 | 350.50 ± 112.74 | 409.08 ± 96.27 |
| **MPV, fL** | 4.85 ± 0.25 | 5.03 ± 0.44 | 4.89 ± 0.32 | 5.16 ± 0.40 | 5.17 ± 0.25 | 5.37 ± 0.44 | 5.40 ± 0.24 | 5.44 ± 0.42 |
| **PDW, CV%** | 15.51 ± 0.27 | 15.73 ± 0.39 | 15.33 ± 0.31 | 15.68 ± 0.32 | 15.60 ± 0.22 | 15.87 ± 0.49 | 15.67 ± 0.30 | 15.83 ± 0.49 |
| **PCT, %** | 0.24 ± 0.07 | 0.19 ± 0.07 | 0.22 ± 0.04 | 0.22 ± 0.02 | 0.24 ± 0.04 | 0.21 ± 0.07 | 0.19 ± 0.06 | 0.22 ± 0.05 |

^1^Data were shown as mean ± SD.

**Supplementary Table S4.** The quantitation of 10 bovine cytokines in the intervention and control groups at -15, 0 and 15 DPI.

| **DPI** | **-15** | | **0** | | **15** | |
| --- | --- | --- | --- | --- | --- | --- |
| **Cytokine** | **Intervention** | **Control** | **Intervention** | **Control** | **Intervention** | **Control** |
| **GM-CSF, pg/mL** | 701.96 ± 600.26^1^ | 399.50 ± 116.74 | 876.89 ± 576.48 | 981.86 ± 1192.24 | 717.10 ± 714.45 | 795.70 ± 905.58 |
| **IFN-γ, pg/mL** | 64362.87 ± 37253.17 | 45887.72 ± 26198.04 | 62129.58 ± 44962.98 | 43191.43 ± 33662.40 | 75794.73 ± 42529.12 | 41065.30 ± 25216.67 |
| **IL-1b, pg/mL** | 53782.87 ± 31824.24 | 51102.42 ± 26313.67 | 60647.91 ± 45128.13 | 54918.05 ± 38512.25 | 61371.71 ± 35901.71 | 54948.05 ± 40775.28 |
| **IL-2, pg/mL** | 51320.86 ± 29283.50 | 46143.05 ± 23418.61 | 57443.32 ± 44038.11 | 55720.24 ± 41278.49 | 56148.10 ± 27921.56 | 57319.84 ± 39318.90 |
| **IL-4, pg/mL** | 72007.52 ± 33675.98 | 72888.37 ± 34320.16 | 80624.66 ± 51125.30 | 70827.88 ± 44724.60 | 77630.81 ± 38971.88 | 73499.64 ± 45518.79 |
| **IL-5, pg/mL** | 48007.59 ± 24601.97 | 45441.51 ± 24868.35 | 53629.07 ± 39835.79 | 51883.49 ± 40467.17 | 51262.49 ± 29964.37 | 52397.92 ± 35458.35 |
| **IL-6, pg/mL** | 75492.72 ± 31785.15 | 73533.29 ± 32077.77 | 85315.69 ± 46679.52 | 73273.74 ± 44045.49 | 83105.28 ± 31790.88 | 72914.73 ± 39580.51 |
| **IL-10, pg/mL** | 91355.54 ± 43529.76 | 75215.19 ± 34820.32 | 88201.49 ± 57079.38 | 63636.42 ± 36719.09 | 108555.82 ± 50554.92 | 69994.84 ± 39582.16 |
| **IL-12p70, pg/mL** | 2535.34 ± 950.60 | 2202.10 ± 353.71 | 3516.12 ± 1273.10 | 3790.69 ± 1537.61 | 4304.16 ± 2483.99 | 2306.02 ± 779.66 |
| **IL-13, pg/mL** | 53175.41 ± 26627.55 | 44841.24 ± 25851.70 | 54179.68 ± 44495.76 | 47384.09 ± 37450.32 | 56159.11 ± 33303.37 | 47098.00 ± 35918.65 |

^1^Data were shown as mean ± SD.

**Supplementary Table S5.** The quantitation of 10 bovine cytokines in the intervention and control groups at 75 and 165 DPI.

| **DPI** | **75** | | **165** | |
| --- | --- | --- | --- | --- |
| **Cytokine** | **Intervention** | **Control** | **Intervention** | **Control** |
| **GM-CSF, pg/mL** | 691.44 ± 245.96^1^ | 609.07 ± 217.08 | 737.32 ± 359.10 | 491.44 ± 236.09 |
| **IFN-γ, pg/mL** | 41554.70 ± 34636.76 | 41290.96 ± 31992.94 | 54033.52 ± 47178.62 | 50604.96 ± 27688.00 |
| **IL-1b, pg/mL** | 41213.01 ± 28547.79 | 49703.90 ± 40587.97 | 47962.17 ± 30575.12 | 56678.11 ± 40353.06 |
| **IL-2, pg/mL** | 44408.54 ± 30655.58 | 49572.48 ± 43523.51 | 43250.01 ± 28951.75 | 49907.93 ± 33370.78 |
| **IL-4, pg/mL** | 56332.51 ± 29874.99 | 64089.90 ± 45238.46 | 69115.58 ± 44862.70 | 79095.65 ± 47419.87 |
| **IL-5, pg/mL** | 41760.18 ± 31090.14 | 45941.35 ± 41421.41 | 42969.41 ± 31569.89 | 44305.66 ± 31729.52 |
| **IL-6, pg/mL** | 63591.27 ± 33473.62 | 64390.72 ± 38474.27 | 64045.99 ± 33662.38 | 78840.15 ± 42471.70 |
| **IL-10, pg/mL** | 55090.44 ± 28530.23 | 56995.41 ± 37284.27 | 70457.04 ± 42054.93 | 69670.31 ± 32342.18 |
| **IL-12p70, pg/mL** | 2966.86 ± 842.10 | 2812.21 ± 1026.97 | 3791.58 ± 1200.86 | 2926.13 ± 711.69 |
| **IL-13, pg/mL** | 37331.42 ± 30165.72 | 38935.22 ± 33729.41 | 40309.72 ± 30029.17 | 37872.49 ± 22808.93 |

^1^Data were shown as mean ± SD.





**Supplementary Figure S1.** Comparisons of CBC parameters between BLV inoculated and uninoculated cows. The full lines and dotted lines indicated the cows in the intervention and control groups, respectively. (A) The number of white blood cell (WBC), 10^9^ cells/L. (B) The number of lymphocyte (LYM), 10^9^ cells/L. (C) The number of monocyte (MNC), 10^9^ cells/L. (D) The number of granulocyte (GRAN), 10^9^ cells/L. (E) The percentage of LYM, %. (F) The percentage of MNC, %. (G) The percentage of GRAN, %. (H) The number of red blood cell (RBC), 10^12^ cells/L. (I) The content of hemoglobin (HGB), g/L. (J) The percentage of hematocrit (HCT), %. (K) The mean corpuscular volume (MCV), fL. (L) The mean corpuscular hemoglobin (MCH), pg. (M) The mean corpuscular hemoglobin concentration (MCHC), g/L. (N) The red blood cell distribution width (RDW), %. (O) The number of platelet (PLT), 10^9^ cells/L. (P) The mean platelet volume (MPV), fL. (Q) The platelet distribution width (PDW), CV%. (R) The percentage of plateletcrit (PCT), %. Data were shown as mean ± SD and the symbols indicated *P* ≤ 0.001 (***), *P* ≤ 0.010 (**) and *P* ≤ 0.05 (*), respectively.
